# Clinical Efficacy and Safety Profile of Suzetrigine: A Novel Non-opioid Analgesic Targeting NaV1.8 Sodium Channel

**DOI:** 10.7759/cureus.96054

**Published:** 2025-11-04

**Authors:** Mamatha M Rejeev, Keechilath Pavithran, Princy Palatty

**Affiliations:** 1 Department of Pharmacology, Amrita Institute of Medical Sciences, Amrita Vishwa Vidyapeetham, Kochi, IND; 2 Department of Medical Oncology, Amrita Institute of Medical Sciences, Amrita Vishwa Vidyapeetham, Kochi, IND

**Keywords:** acute pain management, nav1.8 inhibitor, non-opioid analgesic, sodium channel blocker, suzetrigine, vx-548

## Abstract

Pain management remains a significant clinical challenge due to the safety concerns and addiction risks associated with conventional therapies. The development of selective NaV1.8 sodium channel inhibitors offers a promising non-opioid alternative. This review evaluates the clinical pharmacology, clinical efficacy, and safety profile of suzetrigine (VX-548), a novel selective NaV1.8 inhibitor for treating moderate to severe acute pain. A systematic literature search of major academic databases, clinical trial registries, and regulatory documents was conducted to gather relevant data published between 2020 and 2025. Suzetrigine exhibits high selectivity for the NaV1.8 channel through a novel allosteric mechanism with significant pain relief compared to placebo and comparable efficacy to a standard hydrocodone/acetaminophen combination. The drug displayed a favorable safety profile with a lower incidence of common opioid-related adverse effects, such as nausea and dizziness, and showed no evidence of abuse potential in clinical and preclinical assessments. Suzetrigine represents a significant advancement in acute pain management as a first-in-class selective NaV1.8 inhibitor. Its recent approval by the U.S. Food and Drug Administration for moderate to severe acute pain underscores its potential as an effective non-opioid therapeutic. While its short-term efficacy and safety are well established, future research should focus on its long-term safety profile and its utility across a broader range of pain conditions.

## Introduction and background

Global pain management crisis

The global challenge of pain management extends significantly beyond the inaccessibility of medication, resulting in immense and preventable suffering. It is estimated that 60% of annual deaths in developing nations, approximately 33 million individuals, require palliative care, yet its availability remains critically low. The World Health Organization reports that a large number of people experience untreated moderate to severe pain each year, including 5.5 million terminal cancer patients and one million advanced-stage HIV/AIDS patients. An analysis indicates that opioid pain medications are practically unavailable in more than 150 countries. This includes highly populated nations such as Indonesia, Nigeria, and Russia, where severe scarcity leads to at least 100,000 deaths annually from cancer or HIV/AIDS without adequate pain relief. This deficiency is exacerbated by several constraints. A survey across 40 countries revealed that 29 nations lacked a national palliative care policy, and 33 nations enforced restrictive regulations on morphine prescribing, such as requirements for special doctor licenses or specific prescription forms. These requirements are not mandated by international agreements [1]. In developing nations such as India, the availability of sufficient acute pain management is notably deficient, with less than 30% of patients receiving appropriate relief [2]. In only 45% of hospitals surveyed were anesthesiologists involved in postoperative pain management; in the remaining 55%, non-anesthesiologists, such as surgeons or nurses, were mainly responsible [2].

Current treatment limitations

The use of opioid medications for medical purposes faces substantial barriers, including strict regulations and limited access for both patients and healthcare providers. The regulatory framework established by India's Narcotic Drugs and Psychotropic Substances Act has posed a substantial obstacle to the adequate use of opioid-based treatments in the country. While regulatory changes have improved opioid access for medical needs, concerns persist regarding prescription practices and attitudes among physicians and patients [3]. Opioid prescriptions carry several risks, including the development of dependence and opioid use disorder, along with common adverse effects such as sedation, respiratory depression, confusion, falls, and constipation [4]. Alternative pain management options, such as non-steroidal anti-inflammatory drugs (NSAIDs), also present safety concerns, including gastrointestinal bleeding, kidney complications, and cardiovascular events [5].

The financial impact of inadequate pain management, especially the overreliance on opioid medications, leads to a substantial economic burden on society. In the United States, annual expenses related to Opioid Use Disorder (OUD) were estimated at approximately $35 billion for healthcare services and $23 billion for the criminal justice system, which includes lost productivity due to incarceration. Furthermore, an additional $92 billion is lost annually in productivity due to OUD. This significant financial strain highlights the critical need for safer and non-addictive pain relief options. Economic modeling has demonstrated that effective non-opioid treatments may be more cost-effective than opioid therapy. The main factors driving these long-term costs are the risks of OUD development from short-term opioid use, subsequent increased healthcare expenditures, and higher mortality rates linked to OUD [5].

Sodium channel pain pathophysiology and drug discovery

Molecular Basis of Pain Signaling

Voltage-gated sodium channels are crucial in pain perception, as they are responsible for initiating and transmitting action potentials within neurons. These channels are composed of a large alpha subunit featuring four homologous repeats, each containing six transmembrane segments that form the channel pore and voltage-sensing domains. Among the nine voltage-gated sodium channel subtypes found in humans, NaV1.7, NaV1.8, and NaV1.9 are particularly relevant for analgesia because they are predominantly expressed in peripheral sensory neurons found in the dorsal root ganglion. This specific expression in the peripheral nervous system, with minimal presence in the central nervous system, offers a unique opportunity to develop targeted pain relief that avoids central nervous system-related side effects, such as addiction and dependence, commonly associated with opioids. The validation of these channels as primary targets for pain therapy is directly supported by genetic studies. Gain-of-function mutations in the *SCN9A *gene, which codes for NaV1.7, are linked to severe pain conditions such as primary erythromelalgia, whereas loss-of-function mutations lead to a congenital inability to experience pain. Similarly, functional variations in *SCN10A*, which encodes NaV1.8, are also associated with human pain sensation. This compelling genetic evidence has made these specific channels the focal point of intensive drug discovery efforts [6-9].

NaV1.8 Characteristics

NaV1.8, which is encoded by the *SCN10A *gene, is a tetrodotoxin-resistant channel characterized by distinct biophysical properties that establish it as a vital element in pain signaling pathways. Unlike NaV1.7, which amplifies minor depolarizing currents, NaV1.8 becomes active at more depolarized membrane potentials and exhibits slower inactivation kinetics. Its rapid recovery from the inactivated state facilitates high-frequency repetitive firing of action potentials in dorsal root ganglion neurons. This is a key mechanism underpinning the hyperexcitability associated with both inflammatory and neuropathic pain. These characteristics enable NaV1.8 to remain active and maintain pain signaling, even when other voltage-gated sodium channel subtypes become inactivated. Previous studies on NaV 1.8 have identified a genetic association between *SCN10A *variants and Brugada Syndrome, a cardiac arrhythmia. However, it is now understood that this connection pertains not to the full-length NaV1.8 channel found in neurons but rather to a C-terminal fragment of the protein known as SCN10A short. This short fragment is produced in the heart and enhances the activity of the primary cardiac sodium channel, NaV1.5. Loss of this SCN10A short fragment can lead to cardiac dysfunction. Suzetrigine is a highly selective inhibitor of the full-length NaV1.8 channel and is not anticipated to interfere with this cardiac-specific mechanism [5,7,8,10].

Development History

The development of a successful NaV1.8 inhibitor has faced numerous obstacles, underscoring the significance of suzetrigine's development. Following limited success in clinical trials targeting NaV1.7, research efforts have intensified towards NaV1.8. While early preclinical drug candidates showed promise, none have been commercialized. For instance, A-803467, an early selective NaV1.8 blocker, effectively reduced neuropathic and inflammatory pain in rat models. However, its utility is hindered by poor oral bioavailability and ineffectiveness against postoperative pain. Similarly, PF-04531083 and PF-06305591 demonstrated preclinical efficacy but failed to succeed in early clinical development. Vertex's initial inhibitor, VX 150 (Vertex Pharmaceuticals, Boston, USA), showed potential in Phase II trials for alleviating acute pain after bunionectomy surgery compared to a placebo. However, its development was halted due to insufficient efficacy, as it did not demonstrate a statistically significant improvement compared to hydrocodone/acetaminophen. It is also associated with adverse effects, such as severe headaches. Suzetrigine is a second-generation drug that is a highly potent molecule with an IC50 of 0.27 nM and an exceptional selectivity exceeding 30,000 times for NaV1.8 over other human NaV channels. This high selectivity, coupled with its proven efficacy in Phase III trials for acute pain, established it as the first non-opioid acute pain medication in over 20 years to receive priority review status from the FDA [8,10,11].

Methodology

Search Strategy

**Figure 1:**
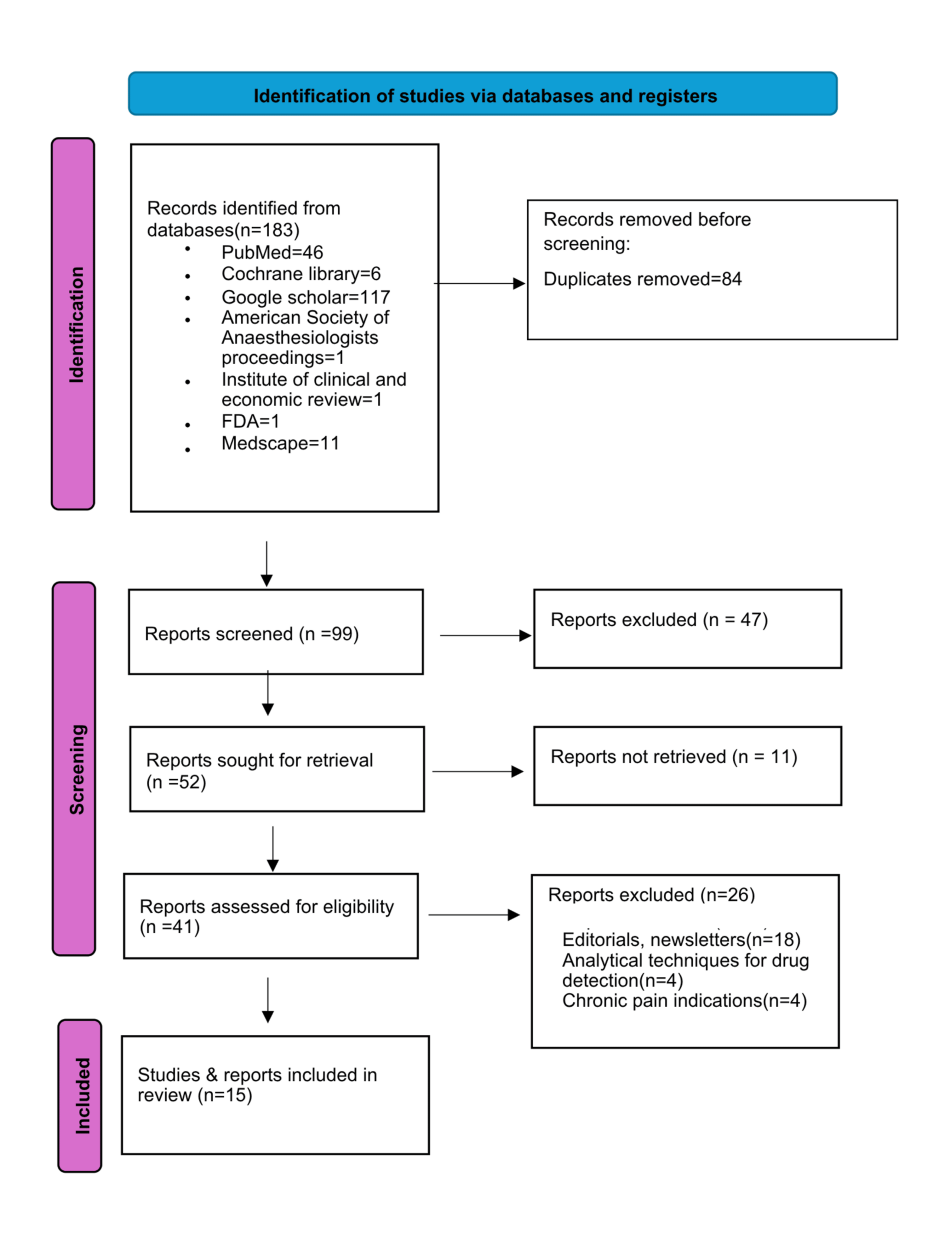
PRISMA flow diagram for search strategy adopted for literature review PRISMA: Preferred Reporting Items for Systematic Reviews and Meta-Analyses [12]

A comprehensive literature search was conducted to investigate the potential utility of VX-548. Databases searched were PubMed, Cochrane Library, Clinicaltrials.gov, Google Scholar, and Medscape, as well as the latest proceedings from the American Society of Anesthesiologists, the Institute for Clinical and Economic Review, and FDA reports. The search terms "VX 548," "Suzetrigine" and "acute pain" were used to identify relevant abstracts and full-text articles. Only articles in English were included. The search strategy and flowchart are presented in Figure 1. The authors thoroughly reviewed all research articles, reviews, and clinical trial reports for inclusion in this comprehensive review.

## Review

Drug overview

Mechanism of Action

Suzetrigine produces pain-relieving effects through a distinct, highly selective, and state-dependent interaction with the NaV1.8 sodium channel. Instead of blocking the channel in the conventional manner of local anesthetics, it functions as a potent allosteric inhibitor. The precise binding site was identified by swapping domains between the NaV1.2 and NaV1.8 channels. This finding revealed that suzetrigine specifically binds to the second voltage-sensing domain of the channel. This site is separate from the central pore, where local anesthetics typically bind. The remarkable selectivity of the drug is attributed to its binding to a unique KKGS amino acid sequence (K = lysine, K = lysine, G = glycine, S = serine) found in the S3-S4 segment's extracellular loop of voltage-sensing domain 2 (VSD2), a motif absent in other human NaV subtypes. By attaching to this site, suzetrigine stabilizes the channel in the closed/resting state. This allosteric action prevents the movement of VSD2, thereby hindering the opening of the channel in response to depolarization and consequently inhibiting the transmission of pain signals. This mechanism leads to an unusual characteristic known as "reverse use dependence," meaning that the drug's inhibitory effect is strongest when the neuron is at rest and can be partially reduced by repeated depolarizations. This behavior contrasts with that of local anesthetics, which exhibit increased binding during channel activation. This novel mechanism allows for consistent tonic inhibition of NaV1.8 across a broad spectrum of voltages and action potential frequencies that may occur in various pain conditions. Furthermore, the chemical structure and binding mechanism of suzetrigine contribute to its exceptional safety and a 31,000-fold selectivity for NaV1.8 compared to all other human sodium channel subtypes. It has also demonstrated selectivity against more than 180 other molecular targets, including 44 linked to abuse potential, with a safety margin exceeding 600-fold relative to the estimated clinical concentrations [9].

Preclinical and Clinical Evidence

Preclinical: A comprehensive series of preclinical investigations involving rats and monkeys confirmed the favorable safety profile and non-addictive characteristics of suzetrigine. Observations in monkeys have demonstrated that suzetrigine did not induce any stimulation or sedative responses, even at exposures up to 15 times the estimated maximum human concentration. No long-term central nervous system (CNS) effects were noted in repeated-dose toxicity studies that spanned up to 26 weeks in rats and 39 weeks in monkeys. A 30-day rat study was performed to directly evaluate physical dependence and withdrawal symptoms. While the morphine positive control group exhibited clear signs of withdrawal upon cessation, the abrupt discontinuation of suzetrigine did not result in any indications of dependence. This observation held true even at exposures up to 52 times the human steady-state maximum concentration (Cmax), a dosage providing over 80% inhibition of rat NaV1.8. To assess the involvement of other sodium channels in heart function, the cardiovascular and respiratory safety of suzetrigine was thoroughly assessed. In telemetered monkeys, a single dose of suzetrigine showed no effects on blood pressure, electrocardiograms, or respiratory parameters. Furthermore, repeat-dose monkey studies extending up to 9 months at exposures equal to or greater than the recommended human dose revealed no quantitative or qualitative ECG changes [9].

Clinical: The promising results from preclinical investigations were directly replicated in human clinical trials, where suzetrigine demonstrated good tolerability and an absence of abuse or addictive potential. This conclusion was drawn from a comprehensive analysis of adverse events observed in three phase III acute pain studies, which collectively involved 2447 participants (1130 treated with suzetrigine for up to 14 days). By utilizing a specially designed MedDRA (Medical Dictionary for Regulatory Activities) query that included almost 200 preferred terms related to abuse potential, the analysis confirmed that the occurrence of these adverse events was minimal and comparable between the suzetrigine and placebo groups. Dizziness, which was the most frequently reported adverse event, appeared at a numerically lower frequency in patients taking suzetrigine (3.8%) than in those taking placebo (6.4%) and hydrocodone/acetaminophen (5.3%). This favorable clinical safety profile, coupled with suzetrigine's mechanism of action, which primarily affects the peripheral nervous system, validates its categorization as a non-addictive analgesic [9].

Efficacy

The efficacy of suzetrigine was primarily evaluated in two pivotal phase III clinical trials: NAVIGATE-1 (NCT05553366) and NAVIGATE-2 (NCT05558410). These randomized, double-blind studies assessed the analgesic efficacy of suzetrigine in postoperative pain management following bunionectomy and abdominoplasty. The trials implemented rigorous inclusion criteria requiring participants to exhibit moderate to severe postoperative acute pain (≥4 on the 0-10 Numeric Pain Rating Scale) [5]. The trials employed a three-arm design comparing suzetrigine (100 mg loading dose, followed by 50 mg every 12 h), hydrocodone bitartrate 5 mg/acetaminophen 325 mg (HB5/APAP325) every six hours, and a placebo. The clinical trials assessed the medication's influence on pain intensity using the Numeric Pain Rating Scale (NPRS) and the time-weighted sum of the pain intensity difference (SPID48) from 0 to 48 h after surgery. A higher SPID48 value indicates a substantial reduction in pain intensity [5].

Suzetrigine vs Placebo:* *The NAVIGATE-1 and NAVIGATE-2 trials showed that patients receiving VX-548 experienced a significantly greater reduction in pain intensity than the placebo groups for both the bunionectomy and abdominoplasty procedures. The analysis demonstrated that including drugs such as ibuprofen for further pain reduction led to higher SPID48 scores in both the VX-548 and placebo groups. Nevertheless, VX-548 still showed a statistically significant advancement in the SPID48 group compared to placebo [5,13].

Suzetrigine vs Hydrocodone Bitartrate/Acetaminophen: In the NAVIGATE-1 and 2 trials, the efficacy of suzetrigine was evaluated as a secondary outcome compared to the hydrocodone bitartrate 5 mg and acetaminophen combination. The imputed analysis of monotherapy data suggested that the hydrocodone/acetaminophen combination was more effective than suzetrigine in the bunionectomy trial, but the difference was not statistically significant when the analysis included treatment with ibuprofen. In the abdominoplasty trial, the observed dissimilarities were not statistically significant. The trials did not appear to be designed as non-inferiority studies, as no non-inferiority margins were specified [5,13].

Suzetrigine vs Higher-Dose Opioids: Higher-dose opioid treatments were pooled using SPID48 values from other clinical trials. These included study arms with oxycodone 15 mg/acetaminophen 650 mg dosed every 12 h and hydrocodone bitartrate 7.5 mg/acetaminophen 325 mg dosed every 4-6 hours. Suzetrigine demonstrated comparable effectiveness in treating moderate to severe acute pain, with no statistically significant differences, although the confidence intervals were wide [5,13].

Suzetrigine vs NSAIDs:* *A network meta-analysis included various formulations and dosages of the nonsteroidal anti-inflammatory drugs, diclofenac, indomethacin, and celecoxib. Based on indirect evidence from this analysis, suzetrigine demonstrated analgesic efficacy similar to that of the included NSAIDs, although the confidence intervals were broad [5].

Safety

Throughout its clinical trial program, suzetrigine exhibited a positive safety and tolerability profile, proving to be comparable to a placebo and potentially even better tolerated than frequently used opioids concerning certain side effects. Suzetrigine appears to be a well-tolerated analgesic agent. The pooled safety profile reported the following percentages of adverse events: nausea (14%), headache (6%), constipation (7%), dizziness (4%), and vomiting (2%). The two serious adverse events observed in the suzetrigine treatment group, namely pulmonary embolism and anemia, were determined to be unrelated to the investigational drug. Approximately 2% of participants terminated suzetrigine therapy due to side effects, including accidental overdose, arrhythmia, nausea, rash, and drowsiness. With the exception of the arrhythmia case, all other adverse events had resolved by the completion of the study. Even though the drug has high selectivity and thereby has reduced CNS and cardiac adverse effects, it has not been studied exclusively in patients with pre-existing cardiac conditions. Hence, it should be used with caution in such populations. Additionally, an intra-abdominal hematoma was reported, which was also considered unrelated to the investigational treatment [5]. Other side effects, such as pruritus, muscle spasms, increased creatine phosphokinase, and rash, have also been reported [14].

Although current short-term data regarding suzetrigine are encouraging, its long-term safety profile, especially concerning cardiac side effects and kidney function, requires continued assessment. A phase II trial investigating suzetrigine for diabetic neuropathy over 12 weeks noted a reduction in creatinine clearance in some patients who received high doses [15]. While this finding suggests a possible risk of kidney injury with extended use, its significance for short-term acute pain management is unclear and warrants further study, particularly in individuals with pre-existing kidney conditions [16].

Pharmacokinetics

Suzetrigine and its active metabolite M6-SUZ exhibited pharmacokinetic characteristics that reached a steady state within three and five days, respectively. At steady state, suzetrigine demonstrated an area under the curve from time 0 to 24 hours​​​​​​​ (AUC0-24h)of 11.5 μgh/mL and a Cmax of 0.62 μg/mL, while the active metabolite M6-SUZ had a higher exposure with an AUC0-24h of 34.7 μgh/mL and a Cmax of 1.5 μg/mL. The median time to reach the peak concentration was 3.0 h for suzetrigine and 10.0 h for M6-SUZ. Both compounds were highly protein-bound (99% for suzetrigine and 96% for M6-SUZ), and suzetrigine had an apparent volume of distribution of 495 L. The mean effective half-lives were 23.6 h for suzetrigine and 33.0 h for M6-SUZ. The primary metabolic pathway for both compounds is via CYP3A, with excretion occurring through both fecal (49.9%) and urinary routes (44%), primarily as metabolites [14].

Precautions, Warnings, and Drug Interactions

Caution is advised when administering it to individuals with severe hepatic impairment, as this may be contraindicated. Additionally, its use in patients with moderate hepatic impairment may increase the risk of adverse events [13]. The concomitant use of suzetrigine with strong CYP3A inhibitors is contraindicated. When used with moderate doses of CYP3A inhibitors, the dose of suzetrigine should be reduced. Dosage alterations may be required when initiating or discontinuing suzetrigine treatment with concomitant CYP3A substrates. Patients using hormonal contraceptives containing progestins other than levonorgestrel and norethindrone should use an additional non-hormonal contraceptive method or an alternative hormonal contraceptive during concomitant use and for 28 days after stopping suzetrigine [14].

## Conclusions

Suzetrigine represents a significant advance in the treatment of acute pain, introducing a new mechanism via selective inhibition of NaV1.8 sodium channels. Its considerable target specificity positions it as a potentially valuable non-opioid analgesic agent. Suzetrigine may be suitable for individuals with a heightened risk of opioid dependence, including older adults undergoing surgery and those with a history of opioid allergy and opioid use disorder who are not currently on opioid therapy. This approach could offer an alternative that enhances the quality of life by mitigating opioid-related adverse effects.

However, significant limitations necessitate further evaluation. The limited scope of existing studies raises concerns regarding long-term safety and efficacy findings, which are predominantly derived from surgical pain models and require exploration in other pain states. Further clinical data are essential to ascertain the long-term impact of the drug on cardiovascular health and its safety profile in patients with renal impairment. Suzetrigine showed efficacy in reducing diabetic peripheral neuropathic pain but was also associated with altered creatinine clearance. Consequently, further research is required to evaluate its safety and utility in chronic pain conditions

The effective integration of suzetrigine into clinical practice will depend on careful patient selection, especially for individuals at an elevated risk of opioid dependence or those with contraindications to nonsteroidal anti-inflammatory drugs. As an innovative therapeutic agent, it has the potential to support opioid-sparing treatment strategies and presents a novel method for managing acute pain.
